# Supplementary material for: Neuronal representation of environmental boundaries in egocentric coordinates
Source: Nat Commun. 2019 Jun 24;10:2772. doi: 10.1038/s41467-019-10722-y (PMC6591168; doi:10.1038/s41467-019-10722-y)
Supplement: Supplementary file 1 — Supplementary Information [file 41467_2019_10722_MOESM1_ESM.pdf]

Supplementary Figure 1

a

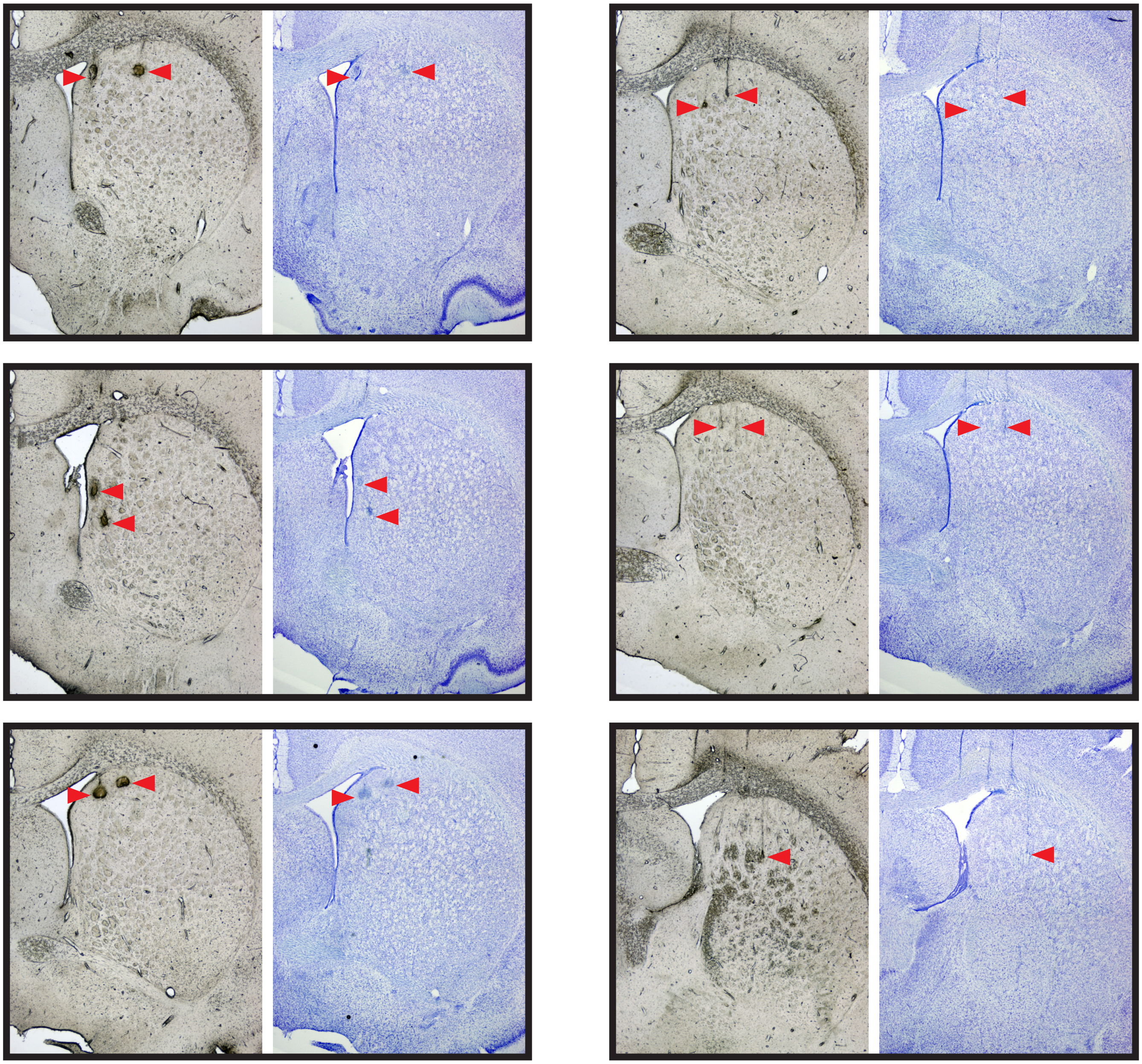

b

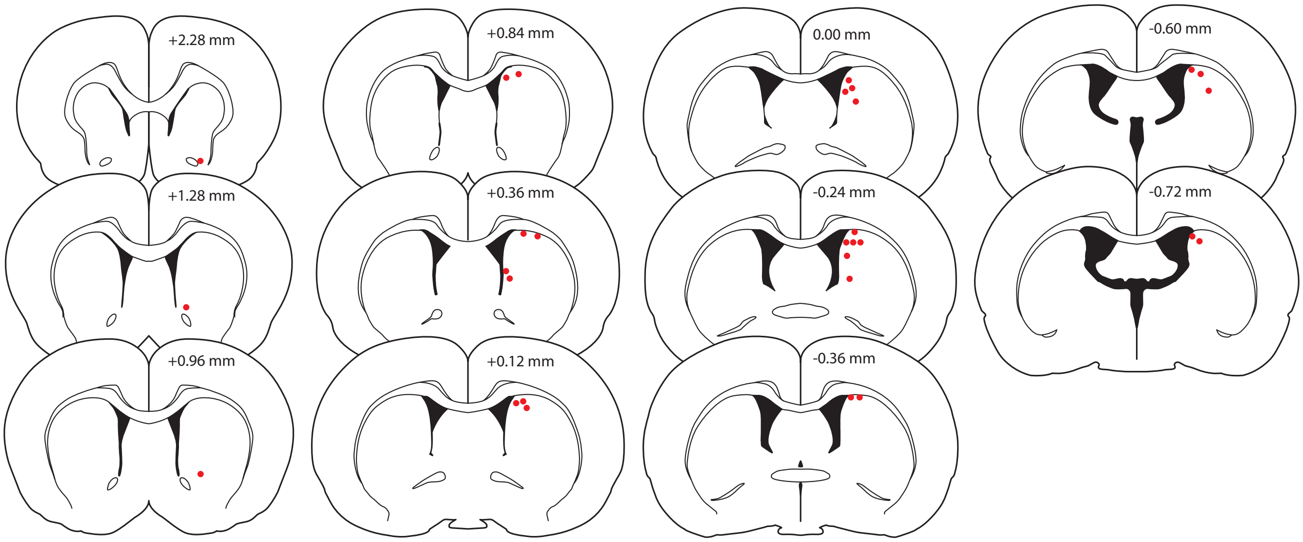

Supplementary Fig. 1 | Recording sites in dorsomedial striatum. a, Six pairs of images of coronal sections show tetrode locations in the dorsomedial striatum. For each pair the image on the left is unstained and the image on the right is Nissl stained. Red triangles point to lesion locations or the end of the tetrode track. **b**, The final position of each tetrode is marked with a red dot on a series of atlas plates reprinted from The rat brain in stereotaxic coordinated, 7th edition, Paxinos, G & Watson, C, Pages 75, 91, 97, 99, 107, 111, 113, 117, 119, 123 and 125, Copyright 2014, with permission from Elsevier.. The distance of each plate relative to bregma is indicated by the distance set within each plate. The final positions of the anterior-most three tetrodes (left column) were approximately 1 mm ventral to the last recorded EBC.

Supplementary Figure 2

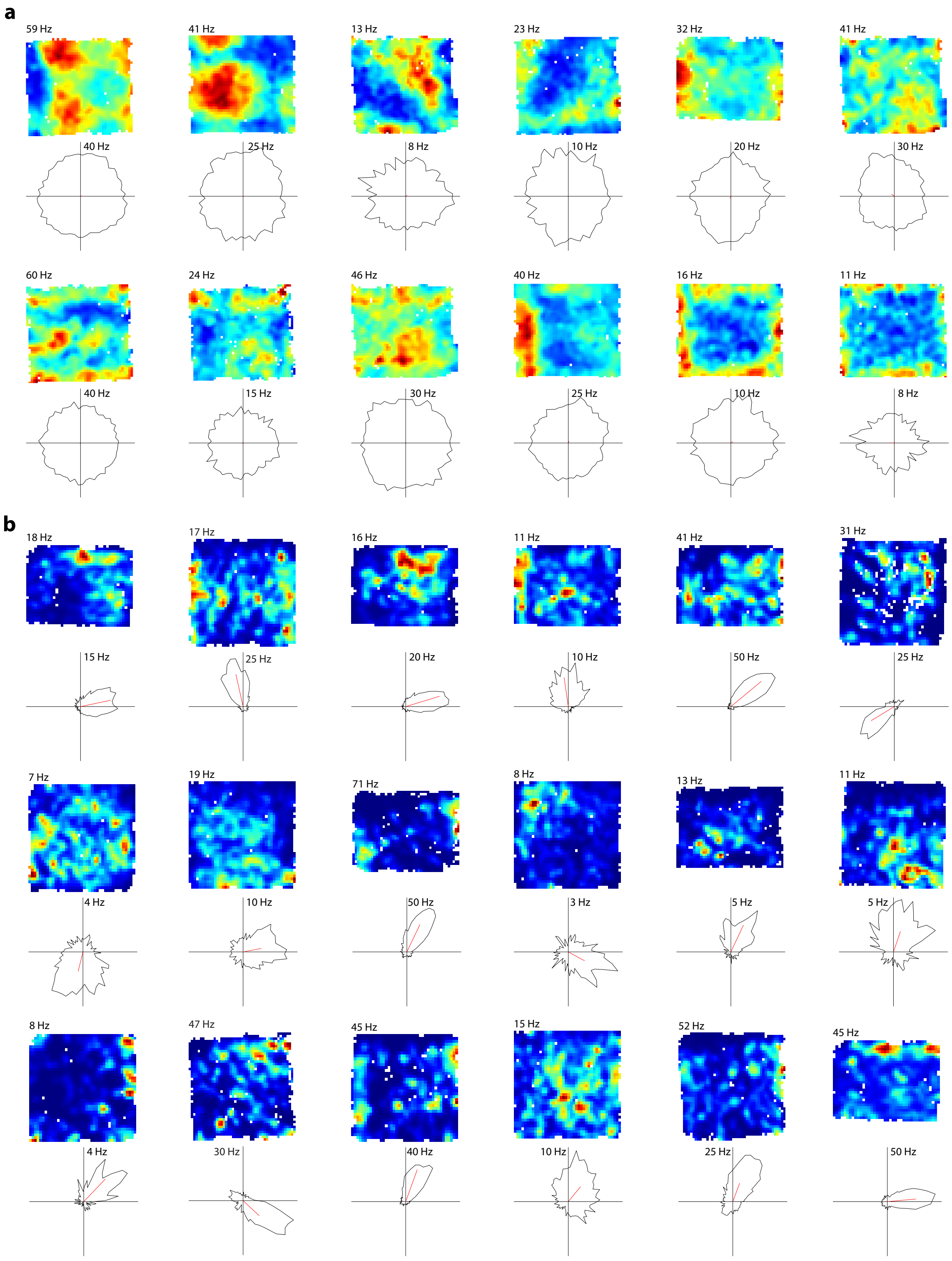

Supplementary Fig. 2 | Allocentric coding in the striatum. a, An allocentric spatial ratemap (*top*) and head direction polar plot (*bottom*) are shown for twelve example allocentric spatial cells. The maximum firing rate for the spatial ratemap is indicated above each plot and the maximum radial firing rate is indicated within each polar plot. **b**, Same plots as in (**a**), but for eighteen head direction cells.

Supplementary Figure 3

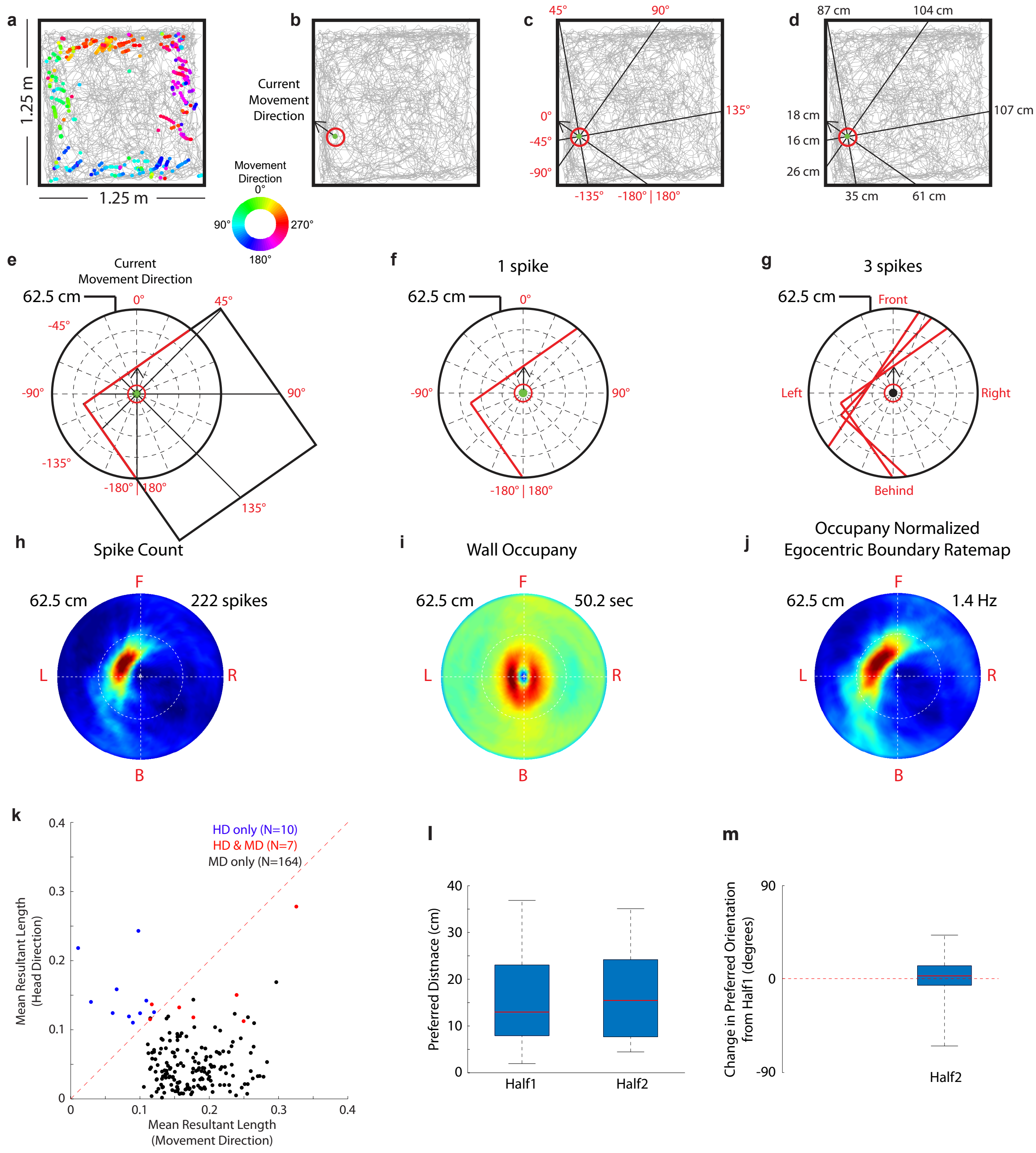

Supplementary Fig. 3 | Egocentric boundary ratemap generation and the use of movement direction instead of head direction. a, The trajectory of the rat through the 1.25 x 1.25 m open field arena is shown with the gray line. The colored circles indicate the location of the rat when the example cell spiked with the color representing the rat's movement direction at the time of the spike. The color legend is set at the bottom right corner of the plot. **b**, A single spike location is highlighted with a red circle along the left hand wall of the arena and an arrow emphasizing the rat's current movement direction. **c**, At the time of each spike the distance to the boundaries of the arena are calculated at all orientations around the rat with just eight orientations shown for clarity and the animal's current movement direction serving as zero. **d**, The distance from the rat to the boundaries is plotted at each of those eight orientations as an example. **e**, The boundaries of the open field are plotted in an egocentric reference frame with the rat positioned in the middle of the polar plot. The radial axis has a maximum of 62.5 cm and the portion of the boundaries that lie within that range are colored red. **f-g**, The boundary positions

are binned across multiple spikes, resulting in a spike count for boundary position (**h**), which is divided by the overall plot of boundary occupancy independent of spikes (**i**) to generate the occupancy normalized egocentric boundary ratemap (**j**). **k**, Scatterplot of mean resultant lengths when using movement direction (MD) versus mean resultant lengths when using head direction (HD) for cells identified as significant EBCs when using either MD or HD. **l**, Box plot of preferred distance where the top and bottom of each box represent the first and third quartile, the red line indicates the median and the whiskers indicate the full range of values for the two halves of the recording. **m**, Box plot of change in preferred orientation from the first half of the recording where the top and bottom of each box represent the first and third quartile, the red line indicates the median and the whiskers indicate the full range of values.

Supplementary Figure 4

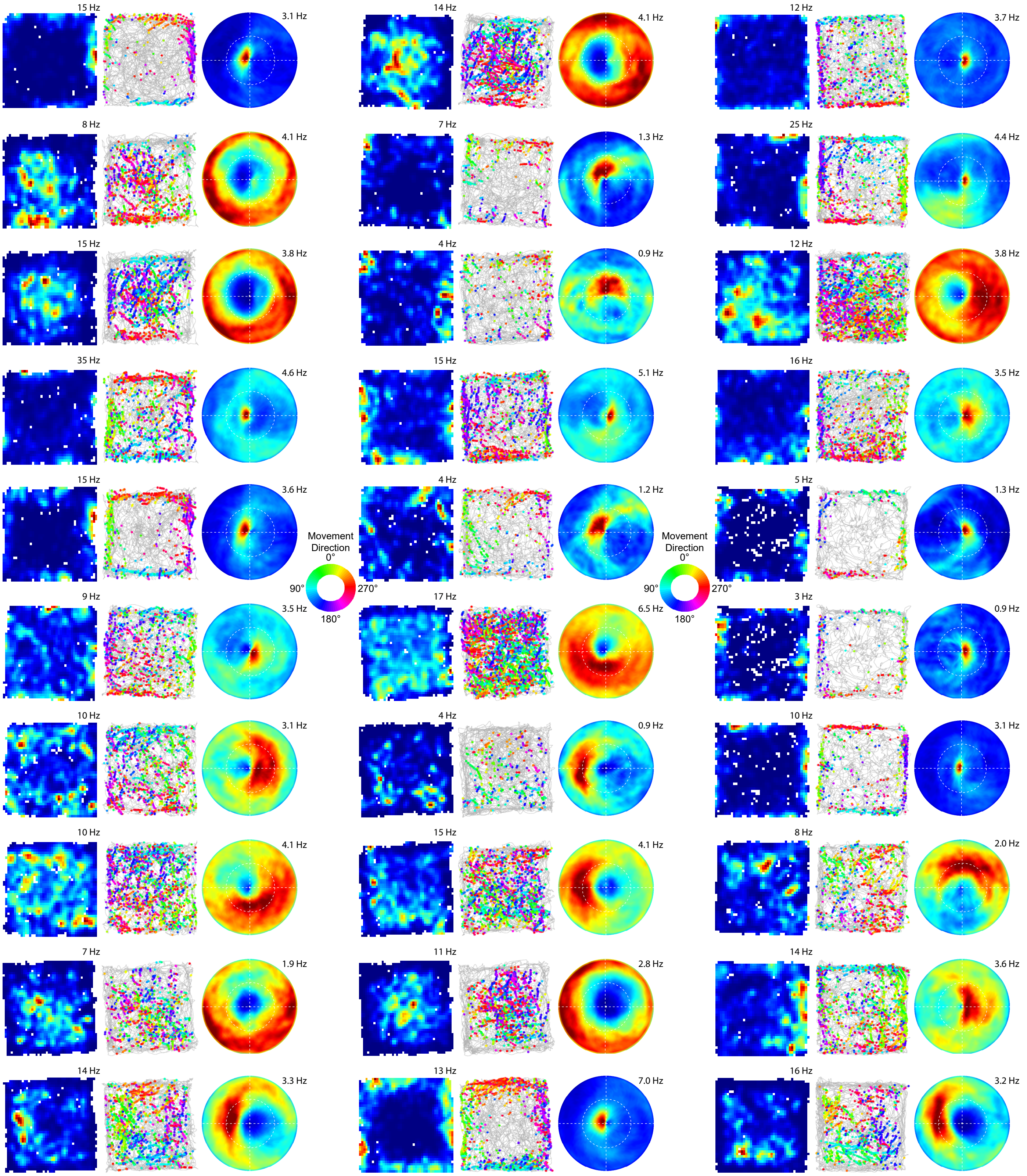

Supplementary Fig. 4 | Additional EBC examples. Three columns of example EBCs illustrate the range of observed EBCs. In each column an allocentric spatial ratemap, a trajectory plot with color-coded movement direction spike locations and an egocentric boundary ratemap are shown for each EBC. The maximum firing rate for the allocentric spatial ratemaps is indicated above the top right corner of each plot, as is the maximum firing rate for the egocentric boundary ratemap indicated above the top right of each egocentric plot. The maximum radial distance for all of the egocentric boundary ratemaps is 62.5 cm. The color legend for the trajectory plots is located in between each column of examples.

Supplementary Figure 5

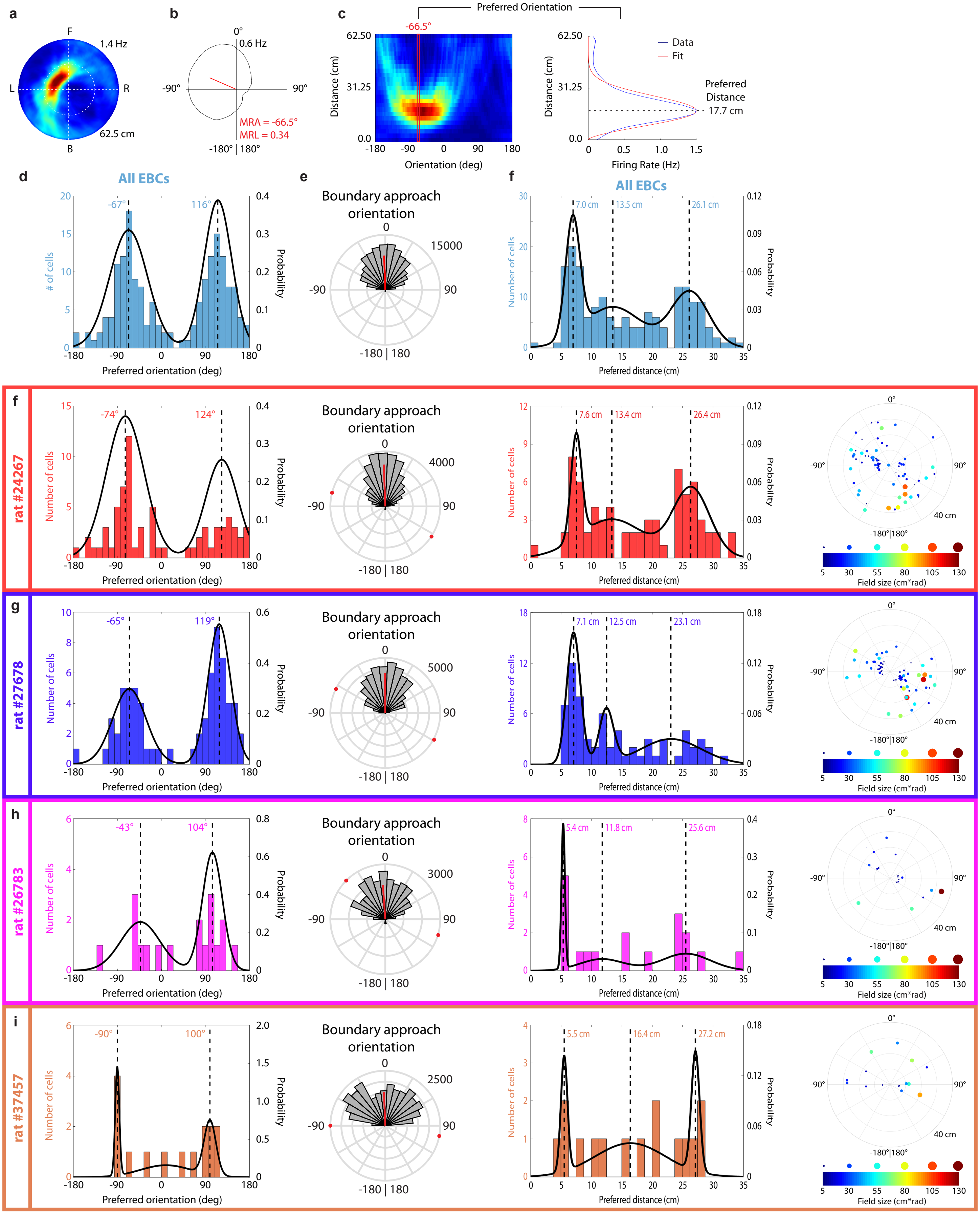

Supplementary Fig. 5 | EBC preferred orientation and distance. a, Example egocentric boundary ratemap (same example as in Fig. 1 and Supplementary Figure 2). The maximum radial distance and maximum firing rate are indicated above and below the plot. **b**, An egocentric boundary direction polar plot is shown that presents the data in (**a**) collapsed across the distance dimension. The mean resultant length (MRL) and mean resultant angle (MRA) are indicated. **c**, On the left is shown an unwrapped egocentric boundary ratemap (from **a**) with the preferred orientation (MRA) highlighted with a red rectangle. The firing rate as a function of boundary distance from the animal (*right*) is plotted (blue) along with the best fit (red) of that data. The peak of the best fit (17.7 cm) was taken as the cell's preferred distance. **d**, Distribution of preferred orientation for all EBCs with the probability distribution function of the mixed Gaussian model overlaid and the peaks of that model marked with vertical dashed lines. **e**, Polar plot of boundary orientation as the rats approached a wall indicating the behavioral approach angle that animals took across all recording sessions. The red line indicates the mean resultant angle. **f**, Same as (**d**), but for the preferred distance of all EBCs. **f-i**, Same plots as in (**d-f**), but for each individual rat and a polar scatter plot of preferred orientation versus preferred distance with field size illustrated by both color and dot diameter (*right*).

Supplementary Figure 6

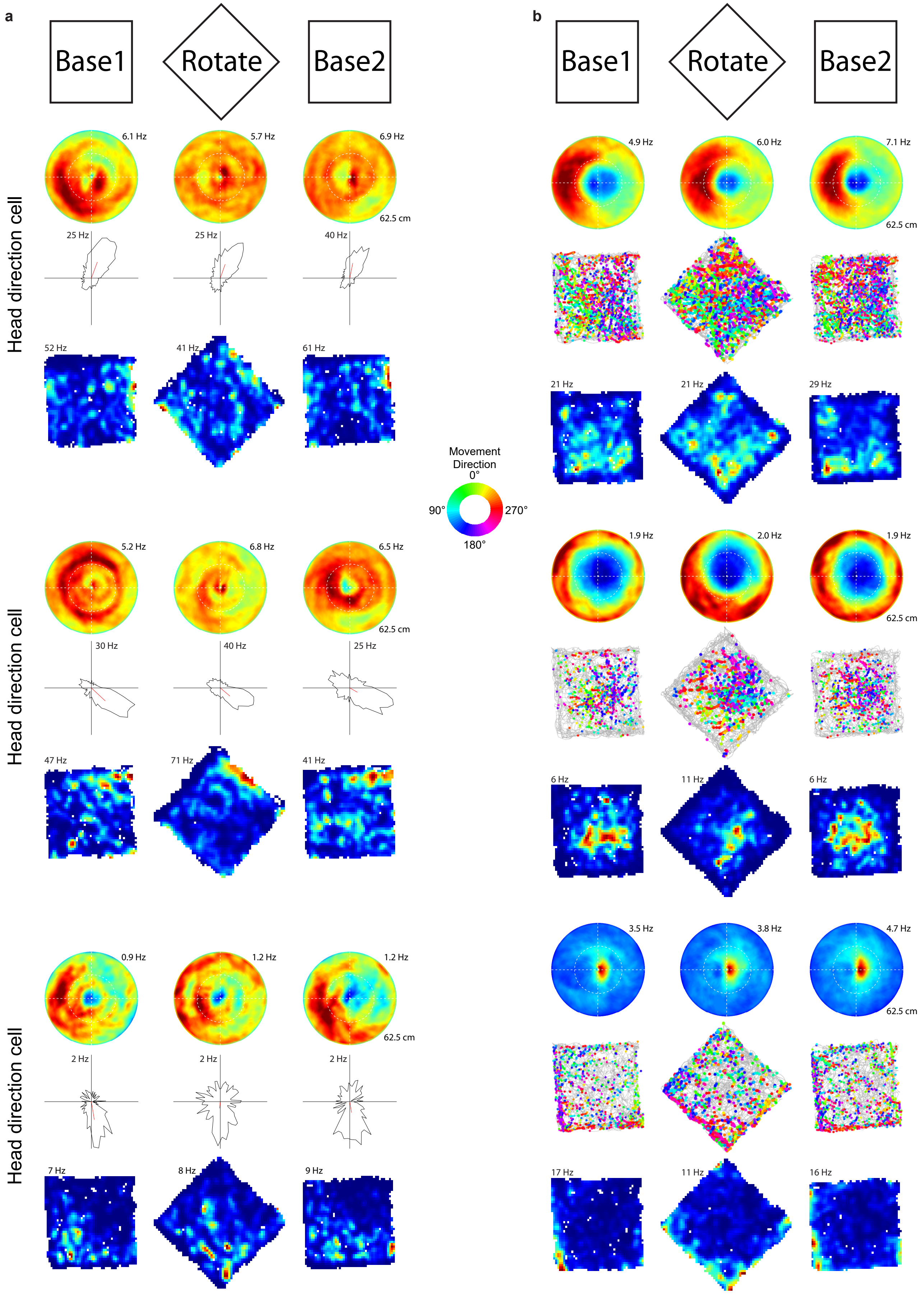

Supplementary Figure 6

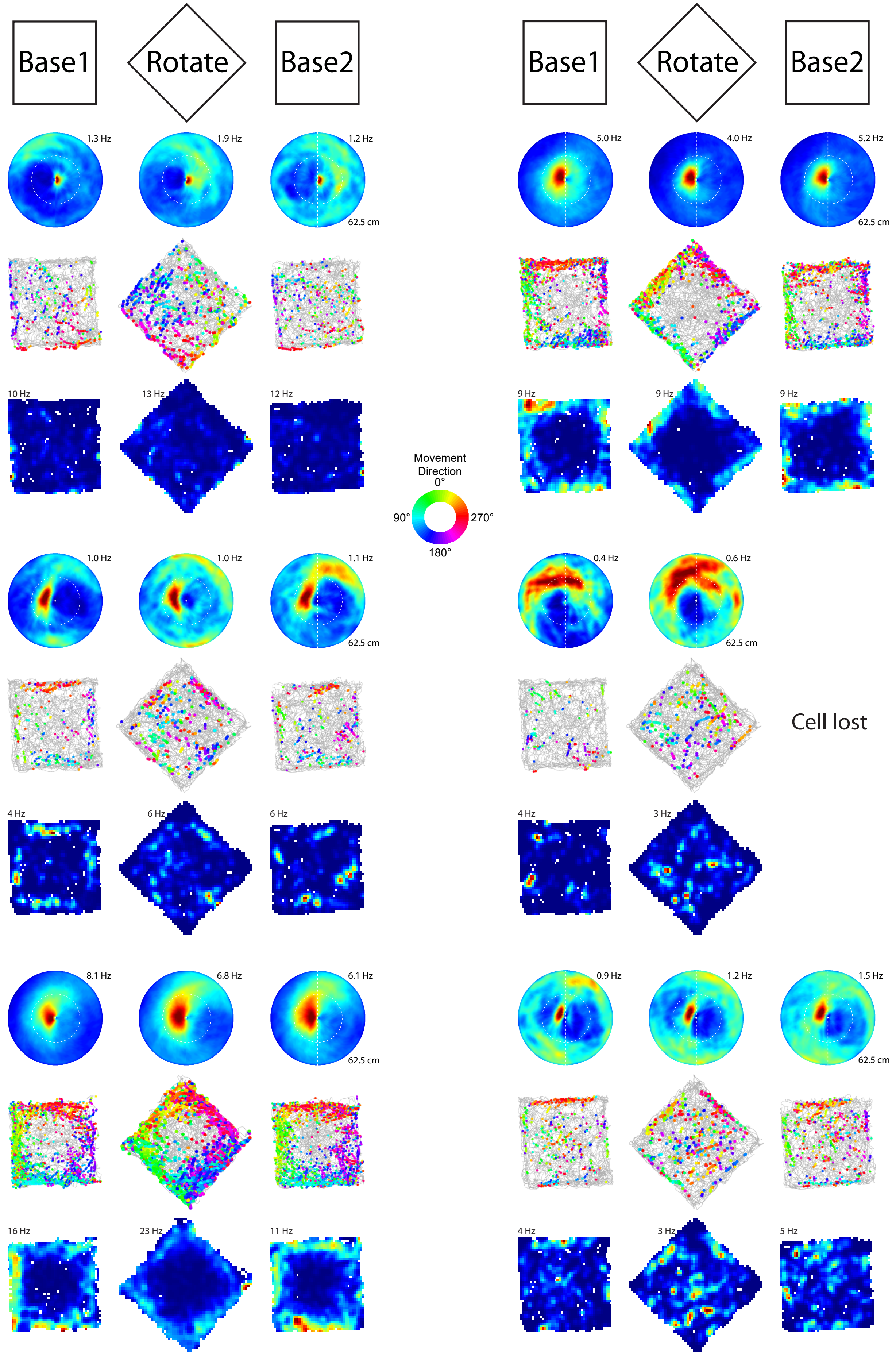

Supplementary Figure 6

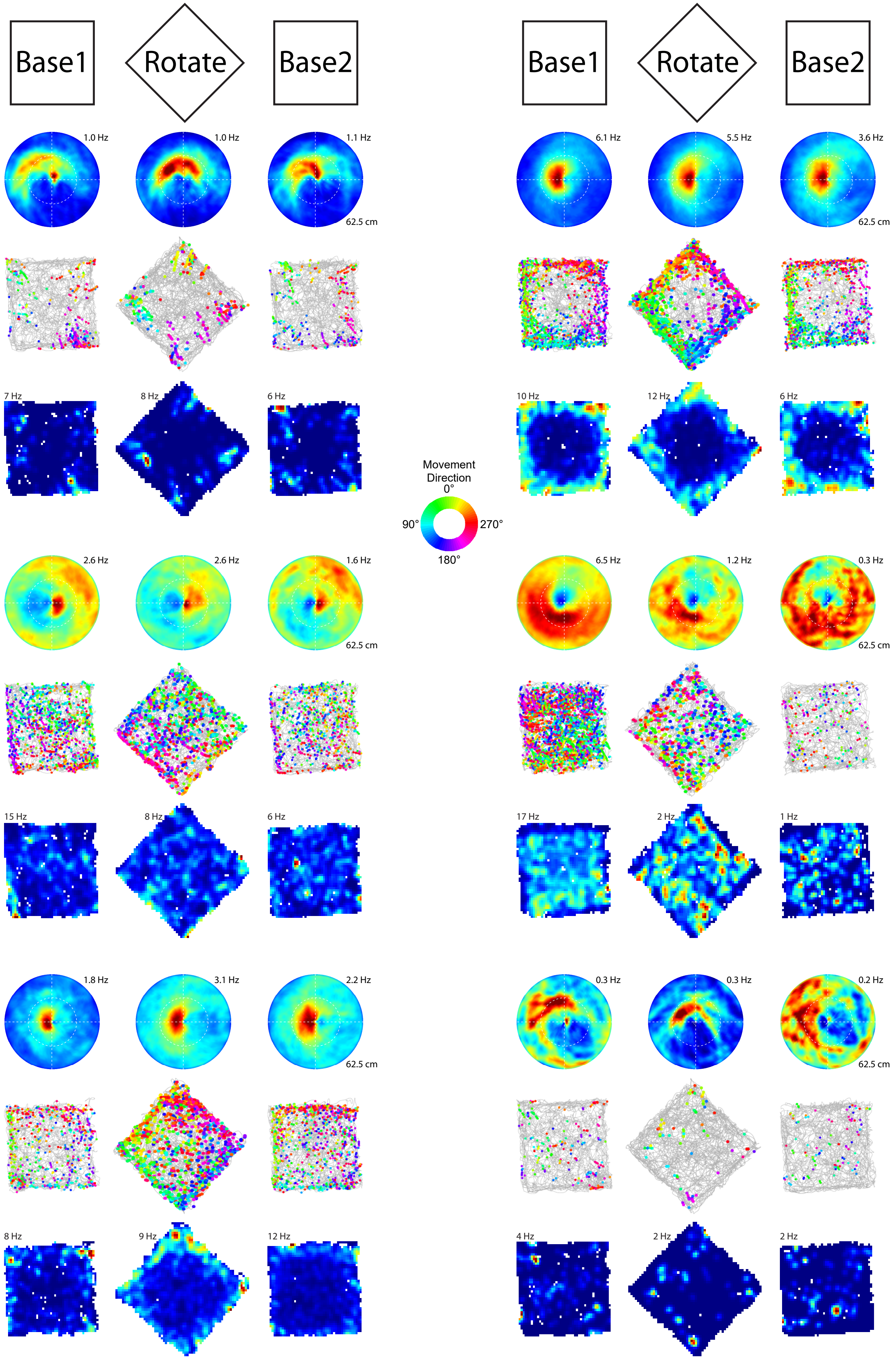

Supplementary Figure 6

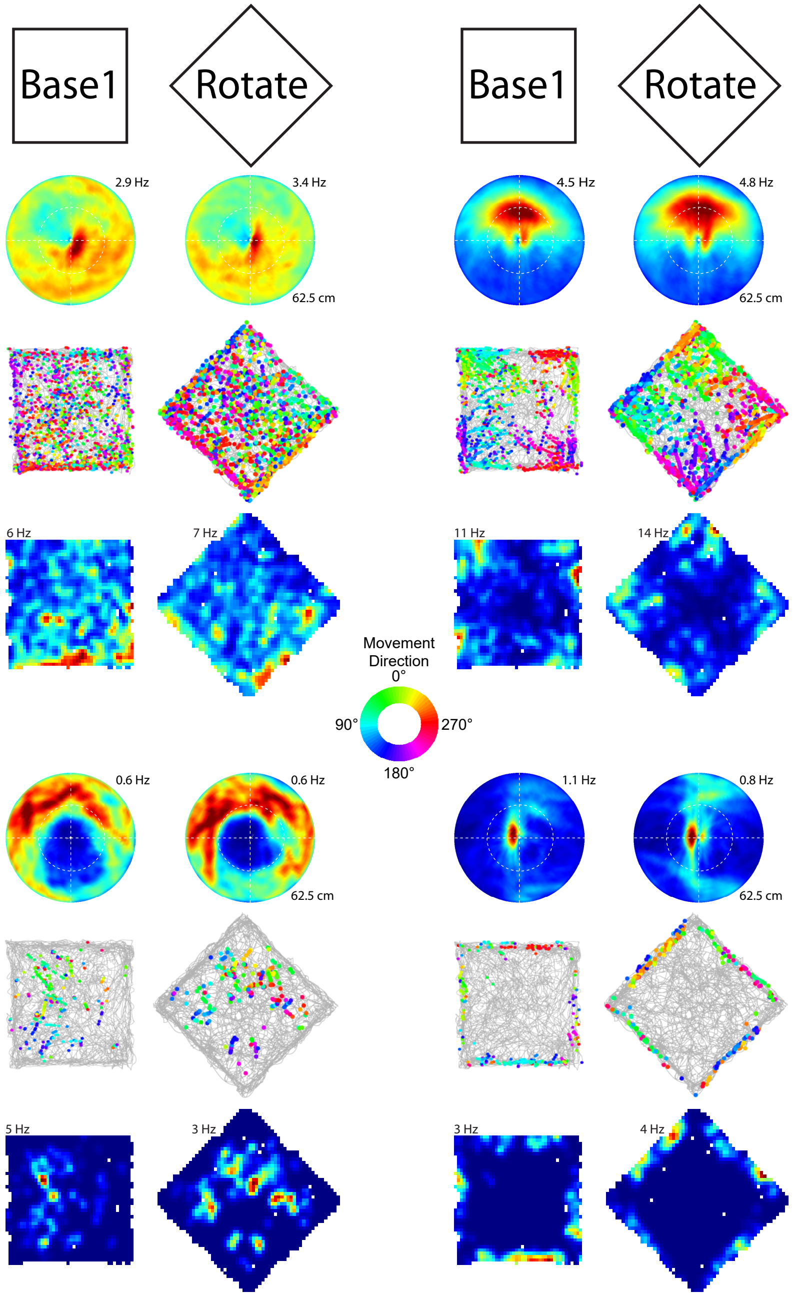

Supplementary Fig. 6 | EBCs and HDCs during open field rotations. a, Egocentric boundary ratemap, head direction polar plot and allocentric spatial ratemap (*top to bottom*) are shown for three example head direction cells during pre-rotation baseline (Base1), environmental rotation (Rotate) and post-rotation baseline (Base2) recordings (*left to right*). **b**, Egocentric boundary ratemap, trajectory plot with movement direction color-coded spike locations and allocentric spatial ratemap (*top to bottom*) are shown during pre-rotation baseline (Base1), environmental rotation (Rotate) and post-rotation baseline (Base2) recordings (*left to right*). The maximum firing rate is indicated above each egocentric boundary and allocentric spatial ratemap and the maximum radial firing rate is indicated at the top of each head direction polar plot. The movement direction color legend is positioned in the middle of each page. Head direction cells maintain their orientation relative to the testing room, while the tuning of EBCs does not change when the open field is rotated indicating that they are not referenced to the distal testing room features, but instead are referenced to the local boundaries.

Supplementary Figure 7

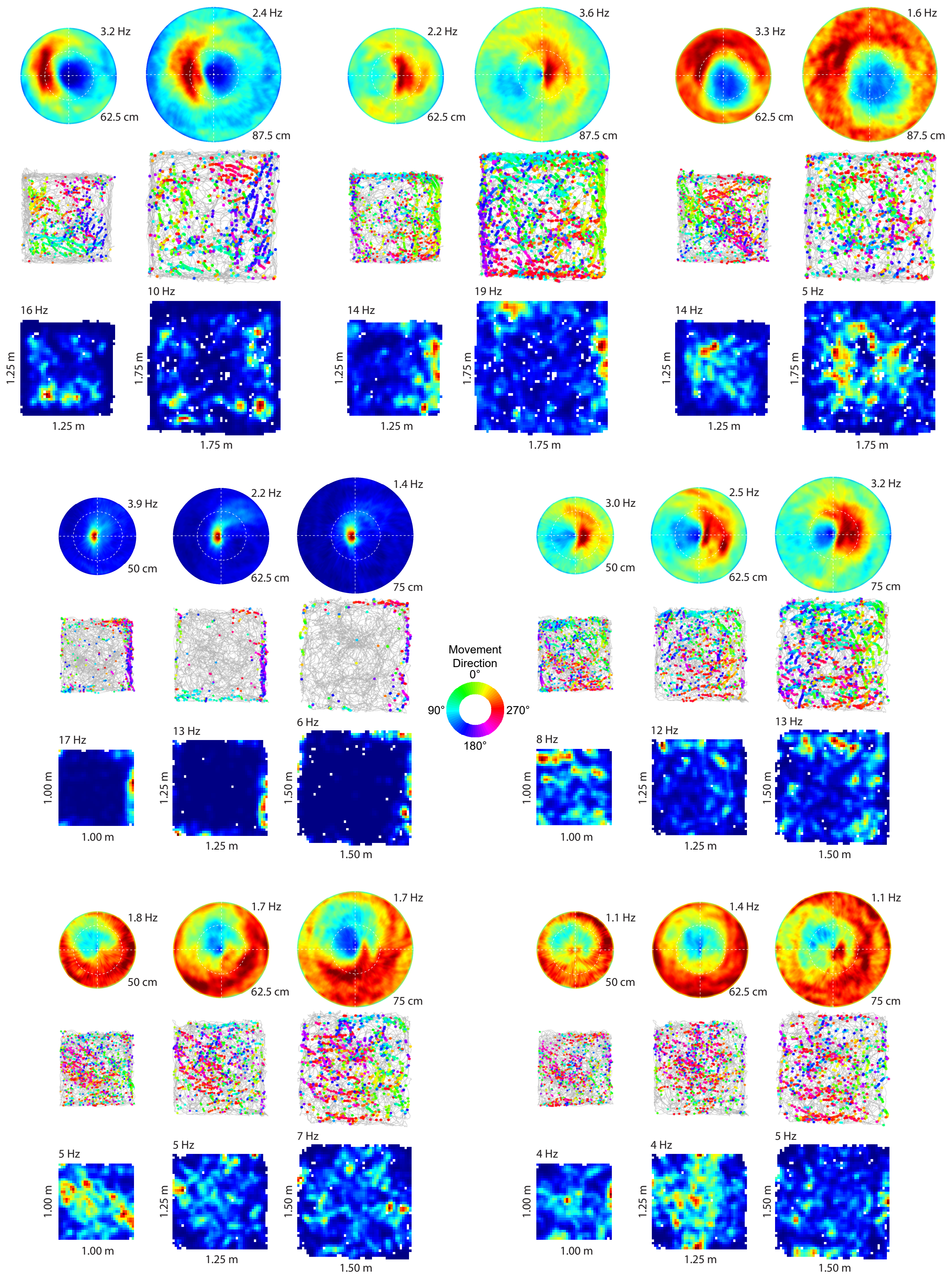

Supplementary Fig. 7 | EBCs do not change scale with changes in the size of the environment. For each cell an egocentric boundary ratemap, trajectory plot with color-coded movement direction spike locations and allocentric spatial ratemap (top to bottom) are shown for recordings in open fields of different sizes. The size of the environment is indicated along the sides of the allocentric spatial ratemaps and the maximum distance of the egocentric boundary ratemaps is scaled accordingly and indicated to the bottom-right of each plot. To facilitate comparisons across environment sizes the dashed white circle within each egocentric boundary ratemap is located at the same distance (31.25 cm) across plots.

Supplementary Figure 8

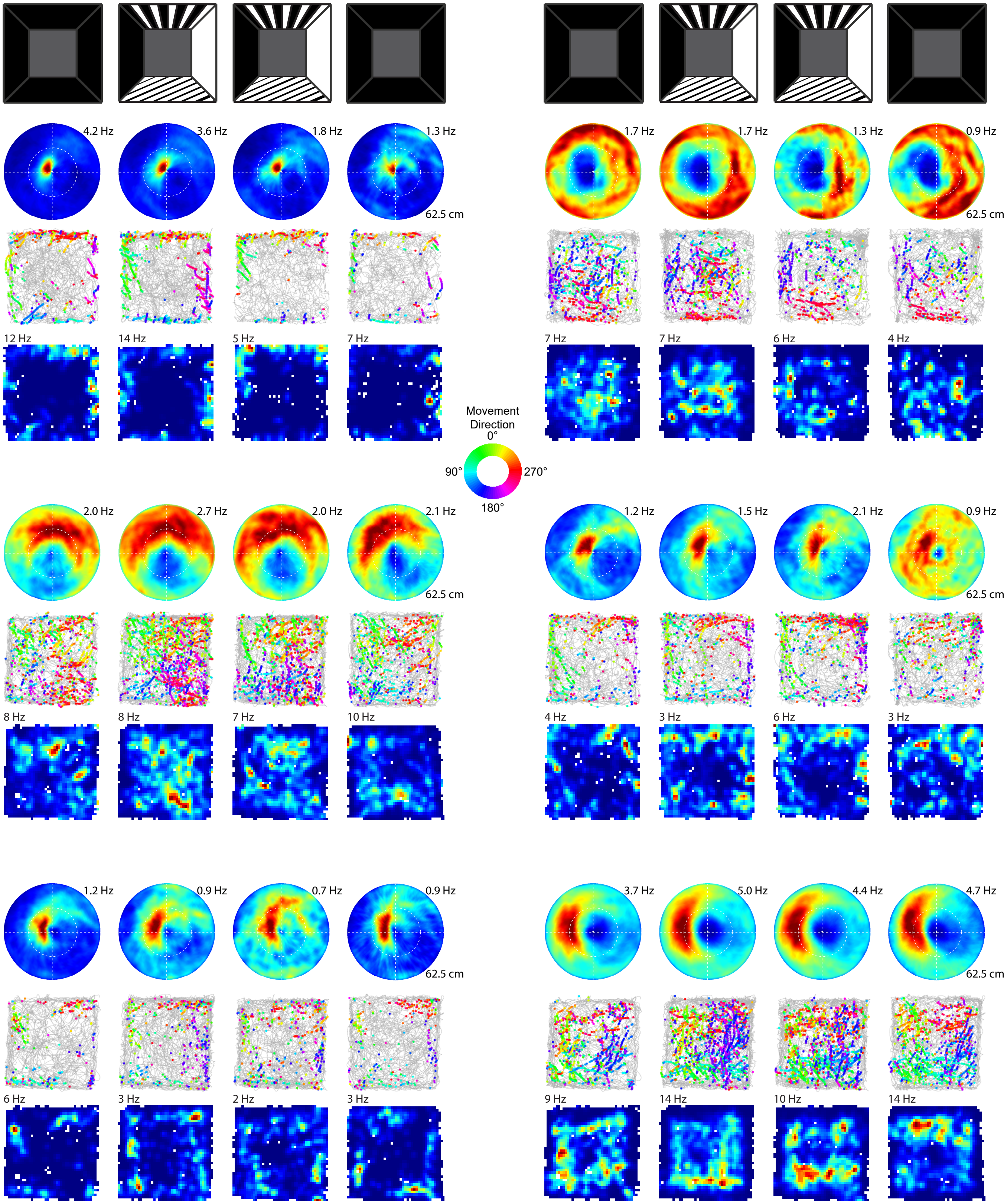

Supplementary Figure 8

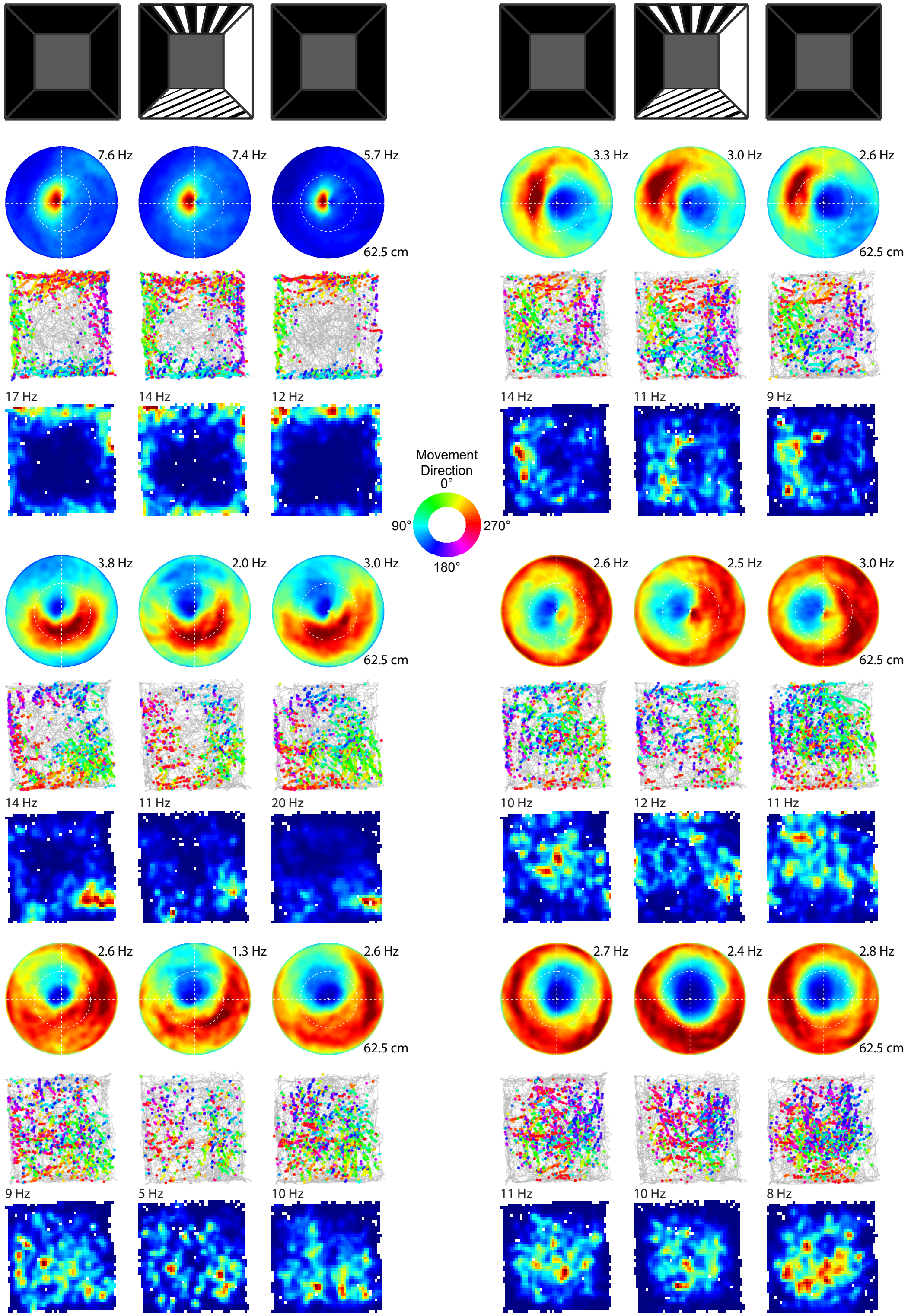

Supplementary Fig. 8 | Invariant EBC response to boundaries varying in visual appearance. For each cell an egocentric boundary ratemap, trajectory plot with movement direction color-coded spike locations and an allocentric spatial ratemap are shown for recordings in an open field with four black walls or the alternate set of visually different walls as depicted at the top of each column. The maximum firing rate is shown to the top right of the egocentric boundary ratemap and top left of the allocentric spatial ratemap. The maximum radial distance of the egocentric boundary ratemaps is shown to the bottom right of the second Baseline recording for each, but is the same for all egocentric boundary ratemaps (62.5 cm). The movement direction color legend is displayed in the middle of the plot.

Supplementary Figure 9

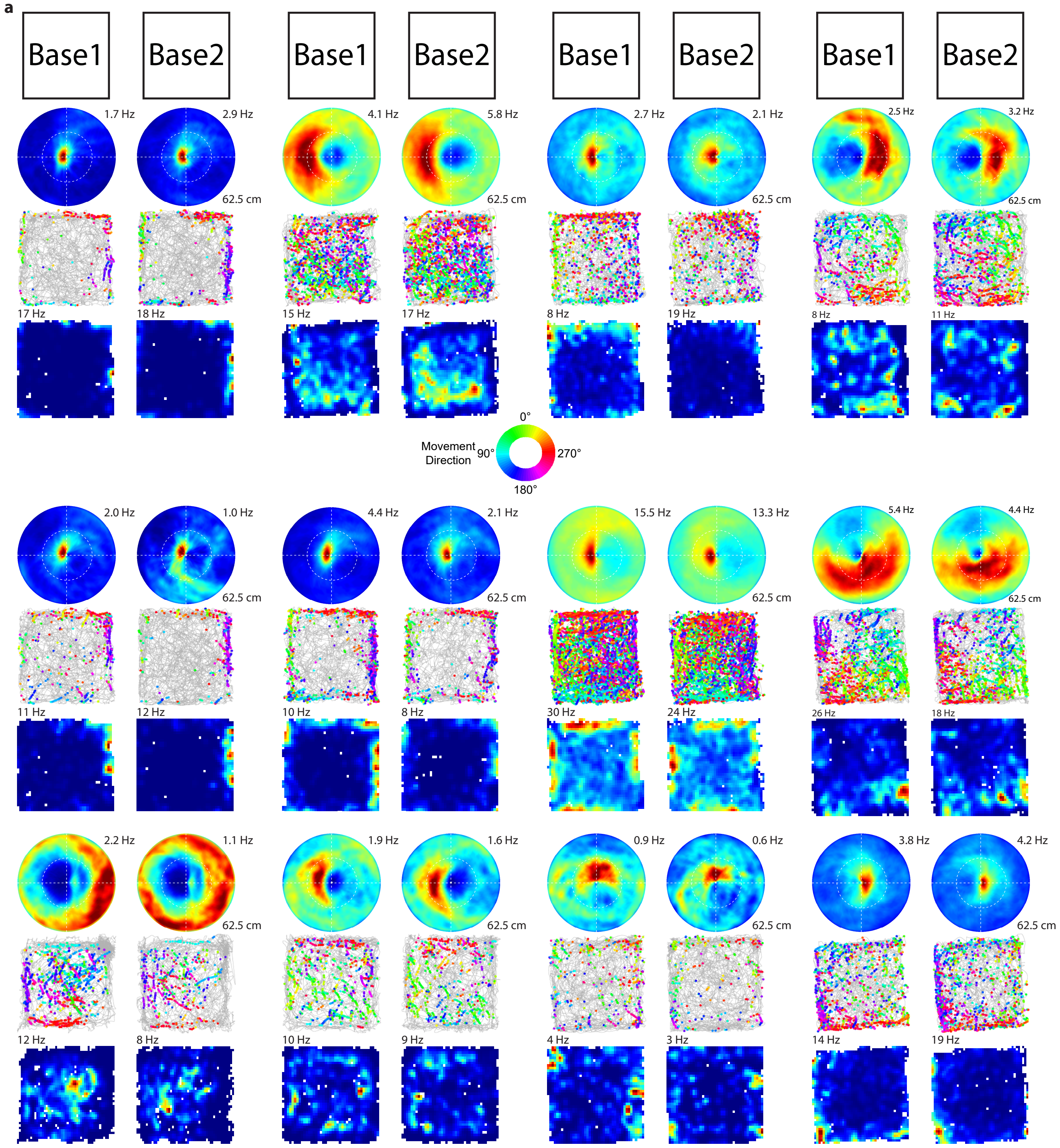

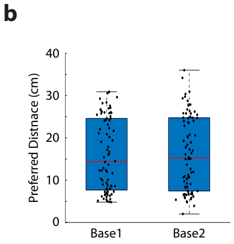

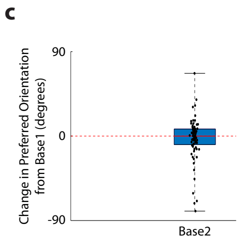

Supplementary Fig. 9 | EBCs respond stably during multiple exposures to the same environment. **a**, Four columns of three EBC examples are shown for two consecutive recordings in the same open field environment. An egocentric boundary ratemap, a trajectory plot with movement direction color-coded spike locations and an allocentric spatial ratemap (*top to bottom*) are shown for the two recordings (*left to right*). The maximum firing rate is shown to the top right of the egocentric boundary ratemap and top left of the allocentric spatial ratemap. The maximum radial distance of the egocentric boundary ratemaps is shown to the bottom right of the second recording for each, but is the same for all egocentric boundary ratemaps (62.5 cm). The movement direction color legend is displayed in the middle of the plot. **b**, Box plot of preferred distance where the top and bottom of each box represent the first and third quartile, the red line indicates the median and the whiskers indicate the full range of values for the two recording (Base1: 25th: 7.67, 50th: 14.41, 75th: 24.59 cm; Base2: 25th: 7.46, 50th: 15.24, 75th: 24.74 cm). **c**, Box plot of change in preferred orientation from the first recording where the top and bottom of each box represent the first and third quartile, the red line indicates the median and the whiskers indicate the full range of values (Base2: 25th: -9.91, 50th: -0.16, 75th: 7.30 degree change from Base1).

Supplementary Figure 10

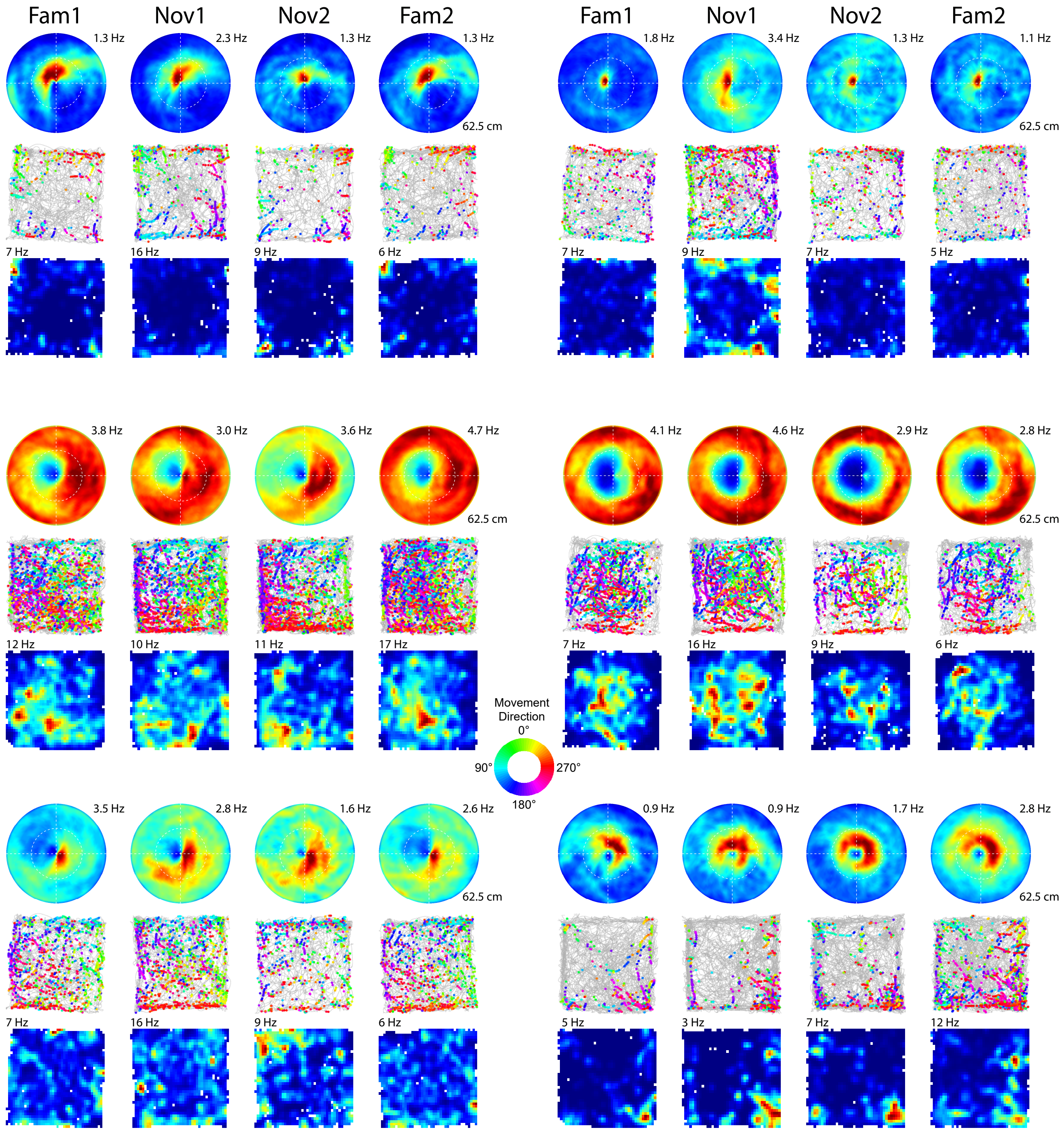

Supplementary Fig. 10 | EBCs do not remap across environments. Two columns of three EBC examples are shown for recordings in a familiar and novel environment. For each EBC an egocentric boundary ratemap, a trajectory plot with movement direction color-coded spike locations and an allocentric spatial ratemap (*top to bottom*) are shown for each of the recordings (*left to right*). The maximum firing rate is shown to the top right and top left of the egocentric boundary ratemap and allocentric spatial ratemap respectively. The maximum firing rate is shown to the top right of the egocentric boundary ratemap and top left of the allocentric spatial ratemap. The maximum radial distance of the egocentric boundary ratemaps is shown to the bottom right of the second familiar room recording, but is the same for all egocentric boundary ratemaps (62.5 cm). The movement direction color legend is displayed in the middle of the figure.

Supplementary Figure 11

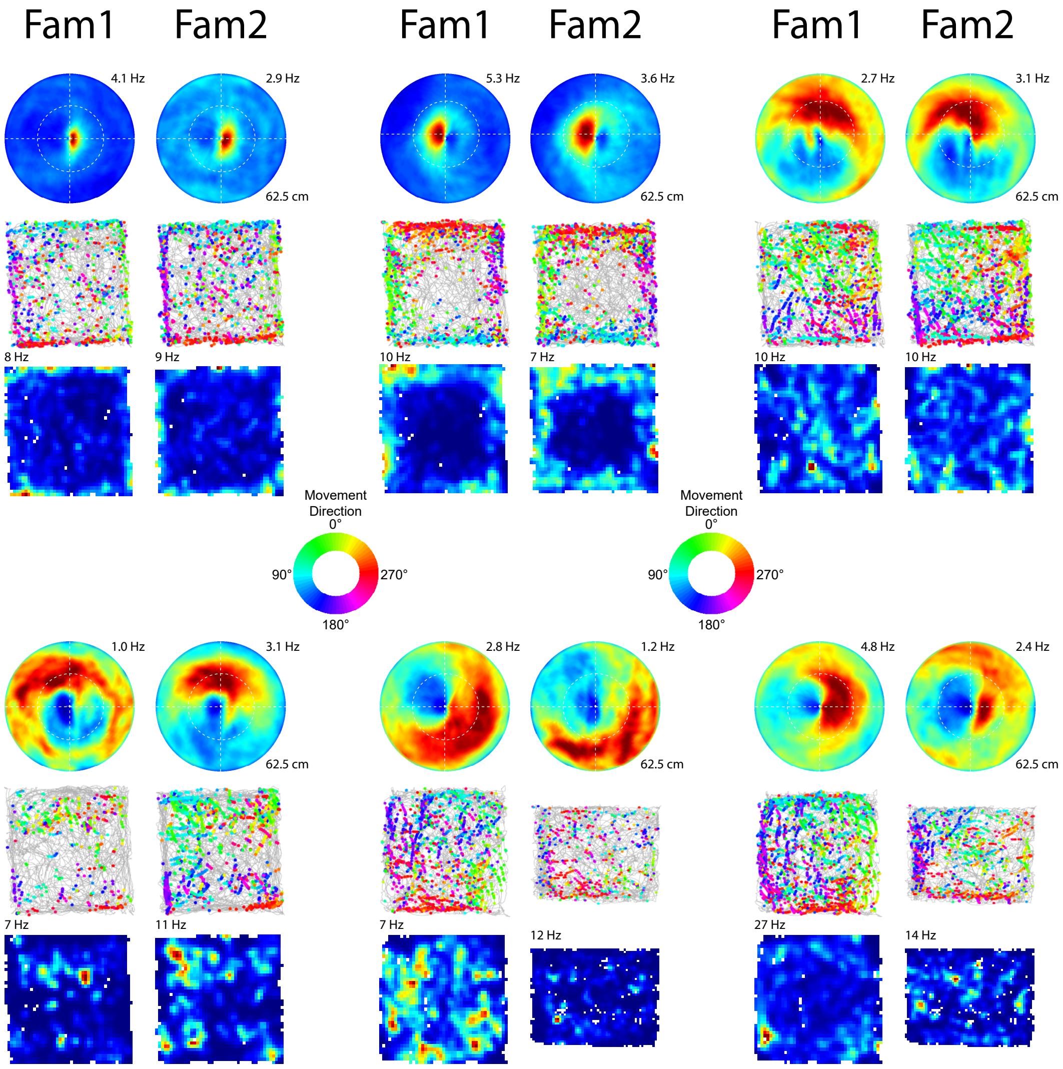

Supplementary Fig. 11 | Additional exposure to multiple environments does not yield different EBC tuning. Three columns of two EBC examples are shown for recordings in two different familiar environments. The second familiar environment is of a different size for two of the examples shown. For each EBC an egocentric boundary ratemap, a trajectory plot with heading color-coded spike locations and an allocentric spatial ratemap (*top to bottom*) are shown for each of the recordings (*left to right*). The maximum firing rate is shown to the top right and top left of the egocentric boundary ratemap and allocentric spatial ratemap respectively. The maximum radial distance of the egocentric boundary ratemaps is shown to the bottom right of the second Familiar recording (Fam2) for each cell, but is the same for all egocentric boundary ratemaps (62.5 cm). The heading direction color legend is displayed in the middle of the plot.
